# Trait Neuroticism and the Nocebo Effect: The Mediating Role of Side‐Effect Expectations

**DOI:** 10.1111/jopy.70056

**Published:** 2026-02-14

**Authors:** Anton Fischer, Li‐Ching Chuang, Nick Augustat, Pia von Blanckenburg, Erik M. Mueller

**Affiliations:** ^1^ Philipps‐Universität Marburg Department of Psychology, Section Personality and Diagnostics Marburg Germany; ^2^ Philipps‐Universität Marburg Department of Psychology, Section Clinical Psychology Marburg Germany

**Keywords:** neuroticism, nocebo effect, side‐effect expectations, somatic symptoms, treatment expectations

## Abstract

**Objective:**

Neuroticism has not only been linked to heightened negative expectations and general somatic complaints but also to increased nocebo responses following medical treatments. Since side‐effect expectations are considered a main driver of nocebo responses, we tested whether side‐effect expectations mediate the neuroticism–nocebo relationship in a pharmacological study of healthy individuals.

**Method:**

Participants (*N* = 275) provided self‐reported neuroticism and pretreatment side‐effect expectations before completing a 2 × 2 factorial design, receiving a placebo or the dopamine antagonist sulpiride (400 mg), which typically does not cause noticeable side effects after a single dose. Regardless of substance, participants received a substance vs. placebo expectation manipulation. We assessed self‐reported somatic symptoms at baseline and during the intervention.

**Results:**

Neuroticism correlated with baseline somatic symptoms and with preintervention side‐effect expectations. Both neuroticism and side‐effect expectations, but not the pharmacological treatment or the explicit expectation manipulation, were associated with nocebo responses, defined as the relative increase in somatic symptoms from before to after the intervention. Crucially, path analyses indicated preintervention expectations partially mediated the neuroticism‐nocebo link.

**Conclusions:**

These results suggest that negative expectations associated with neuroticism contribute to nocebo responses, potentially creating a feedback loop whereby negative expectations heighten side effects, reinforcing negative expectations in future treatments.

## 1 Introduction

### 1.1 Neuroticism, Negative Expectations and General Health Complaints

Neuroticism is a personality trait with remarkable public health significance, with decades of research showing that higher neuroticism levels are associated with a more negative outlook on life, as well as poorer mental and physical health (Lahey 2009).

The trait reflects a general disposition toward negative emotionality and emotional instability. More precisely, it is the personality factor covering the covariance between items assessing anxiety, depression, irritability, anger, frustration, and hostility (Costa and McCrae 2008; DeYoung et al. 2007). While different labels have been used, a very similar broad negative affect related trait (NART) (Brandt and Mueller 2022) can be found in almost every major personality model from early to contemporary biological approaches, in factor‐analytic models as well as in clinically driven personality frameworks (Cloninger 1986; Costa and McCrae 1992; Eysenck 1944; Gray and McNaughton 2000; Tellegen and Waller 2008).

Neuroticism is robustly associated with negative beliefs and expectations about oneself and the world. Individuals high in neuroticism endorse negative core beliefs, exhibit lower self‐esteem and self‐efficacy (Amirazodi and Amirazodi 2011; Otani et al. 2020; Piechurska‐Kuciel 2021), view past experiences more negatively, and anticipate future outcomes more pessimistically (MacLeod 1996; Quoidbach et al. 2008). In general, neuroticism is associated with pervasive negative expectations across broad life domains (Fischer and Mueller 2025).

Furthermore, neuroticism is associated with a tendency to construe events negatively, to process negative information selectively, to use maladaptive emotion‐focused coping strategies, and mood spillover, etc., all contributing to elevated daily negative affect, thereby sustaining neuroticism over time (Suls and Martin 2005). Similar processes likely maintain neuroticism‐associated negative expectations (Fischer and Mueller 2025).

The association between neuroticism and heightened somatic complaints is likely also sustained by negatively skewed information‐processing biases, such as somatosensory amplification, somatic misattribution, and increased engagement with bodily sensations (Denovan et al. 2019; Gaggero et al. 2022; Wise and Mann 1994).

Taken together, perceptual and interpretive biases play a key role in driving negative expectations and elevated somatic symptoms among high‐neuroticism individuals (Lahey 2009; Van Den Bergh et al. 2017).

### 1.2 Neuroticism, Negative Treatment Expectations and the Nocebo Effect

Given the robust association of neuroticism with heightened somatic complaints and negative expectations across life domains, neuroticism has also long been discussed as the primary personality factor predisposing individuals to negative outcomes after medical treatments that cannot be explained by their pharmacologic effects or after sham interventions, that is, to nocebo responses (Barsky 2002).

However, empirical evidence is somewhat inconclusive here: A systematic review by Kern et al. (2020) found only small, context‐dependent direct associations between neuroticism and heightened nocebo responses. At the same time, the empirical foundation may be too weak to close the subject, given that only *k* = 10 studies with a moderate median sample size of *n* = 52 had explicitly investigated the link between neuroticism and heightened side effects (Kern et al. 2020). In light of studies showing that correlations in personality research require sample sizes in the range of *n* > 200 in order to stabilize (Schönbrodt and Perugini 2013), larger samples are needed.

Meanwhile, experiments show that negative treatment expectations influence nocebo responses and can also alter side‐effect profiles for active substances (e.g., Benedetti et al. 1997; Bingel et al. 2011). Consequently, negative expectations are increasingly viewed as the central mechanism linking both contextual and personality factors to nocebo responses (Rief and Petrie 2016). In support of an indirect effect of neuroticism on nocebo responses, a multisite study (*n* = 748) found that higher neuroticism predicted side‐effect expectations across diverse medical interventions (Basedow et al. 2023).

### 1.3 Current Study

In sum, prior research demonstrates that neuroticism is strongly associated with somatic complaints and generalized negative expectations. Evidence on a direct link between neuroticism and heightened nocebo responses remains somewhat inconclusive; however, with mostly small studies examining the effect. The present study starts here.

We conducted a secondary analysis of a large, published dataset (see Chuang et al. 2025) for an exploratory test of five hypotheses. Neuroticism is associated with elevated somatic symptoms in general (hypothesis 1). Neuroticism is linked to a stronger increase of somatic symptoms after a medical intervention relative to baseline (hypothesis 2). Neuroticism predicts side‐effect expectations with regard to this intervention (hypothesis 3). Higher side‐effect expectations predict higher increases of somatic symptoms after this medical intervention (hypothesis 4). The relationship between neuroticism and the increase in somatic symptoms after the intervention is (partially or fully) mediated by side‐effect expectations (hypothesis 5).

## 2 Materials and Methods

Data for this publication was collected as part of a larger project on treatment expectations, reward learning and affect (see Section 2.2 and Chuang et al. 2025 for details). Consequently, only a subset of instruments used in the procedure were utilized for this analysis (see Section 2.3). Given that data collection had already been completed prior to the conceptualization of the current study, the specific hypotheses and analytical strategy were not preregistered.

### 2.1 Participants

Right‐handed participants of any gender aged 18–65, not meeting any one of the following exclusion criteria, were eligible to participate: A positive pregnancy test, current major medical conditions, prescription medication use within the past 3 months, a BMI below 19 or above 30, suspected DSM‐IV Axis I disorders as assessed by mini‐DIPS (Margraf and Cwik 2017), regular cigarette smoking, alcohol or drug abuse, high caffeine intake (> 8 cups a day), and others (for a detailed table all exclusion criteria, see Table S1).

A total of *N* = 297 participants were assessed, with *n* = 285 participants completing all relevant questionnaires as well as the experimental manipulation. Participants with extreme values—that is, exceeding three times the interquartile range in any variable—were excluded (*n* = 10) to ensure reliable metric correlations and regression estimates, as these measures are sensitive to extreme values (Barnett and Lewis 1994; Wilcox 2012b, Wilcox 2012a; Table S2). Thus, the final analyzed sample comprised of *n* = 275 participants with 133 identifying as female, 139 as male, and 3 as gender diverse (Age: *M* = 24.40, SD = 3.65). The study was approved by the Ethics Committee of Marburg University's Medical Department, following the Declaration of Helsinki.

### 2.2 Study Design

The study followed a fully crossed 2 × 2 experimental design with the factors Substance and Expectation. With regard to the factor Substance, participants either received 2 × 200 mg sulpiride pills or two placebo pills, with equal probabilities for both conditions (for details on the pharmacological substance, see Section 2.4). Independently of the substance manipulation, participants' expectations were manipulated using a standardized verbal instruction alongside the substance administration, so that participants were either informed that they received two placebo pills (low treatment expectation) or two sulpiride pills (high treatment expectation), again with equal probabilities for both levels (for details, also see Section 2.4). Sample sizes were as follows: sulpiride/high‐expectation, *n* = 71; sulpiride/low‐expectation, *n* = 68; placebo/high‐expectation, *n* = 70; placebo/low‐expectation, *n* = 66. Data collection took place between December 2021 and September 2023.

### 2.3 Procedure

Participants were recruited via flyers, posters, personal ads, and the internal participation recruitment system at Marburg University. Participants received either 10 course‐credit units or 130 €, with the opportunity to earn up to an additional 20 € based on task performance. After a telephone screening to check for inclusion and exclusion criteria, eligible individuals received a study invitation mail, also providing general information on the 400 mg dosage of sulpiride, including potential main and side effects.

Invited participants were asked to arrive on an empty‐stomach at 8 a.m. Upon arrival, participants filled out an informed consent sheet and completed a battery of questionnaires, including side‐effect expectations and general somatic symptoms ratings. Female participants were required to take a pregnancy test, which had to be negative to avoid any potential risks due to sulpiride intake during pregnancy. Side‐effect expectations were measured by the Generic rating scale for previous treatment Experiences, treatment Expectations and treatment Effects (G‐EEE; Rief et al. 2021; see Section 2.4). General somatic symptoms were measured by the General Assessment of Side Effects questionnaire (GASE_Baseline; Rief et al. 2011; see Section 2.4).

At approximately 9 a.m., a medically trained person—a medical doctor or nurse—took saliva and blood samples (not relevant for this publication) and subsequently administered the substance (substance manipulation) alongside the standardized verbal instruction (expectation manipulation) (see Section 2.4 for details). Following the experimental manipulation, a standardized breakfast was provided.

After breakfast, an additional blood sample was collected and participants filled out the German version of the Big Five Aspect Scales (BFAS‐G; Mussel and Paelecke 2018; see Section 2.4). Following that, EEG electrodes and peripheral physiological devices were attached and participants subsequently completed different experimental tasks: the Alternative Uses Task (Guilford 1967), a probabilistic reinforcement learning task (Frank et al. 2004), the Effort‐Expenditure for Rewards Task (EEfRT; Treadway et al. 2009), and a mood induction task (Chuang et al. 2024).

At the end of the study, participants filled out a last set of questionnaires, including the General Assessment of Side Effects questionnaire post treatment (GASE_Post; Rief et al. 2011; see Section 2.4). Participants typically finished with the experiment around 2 p.m., resulting in an overall study duration of approximately 6 h. For an in‐depth description of the procedure, see (Chuang et al. 2025).

### 2.4 Materials

#### 2.4.1 Substance Manipulation

As stated above, either 2 placebo pills or 2 × 200 mg sulpiride pills with identical appearance were administered in a standardized way to the empty‐stomached participant. Sulpiride is a selective dopamine D2‐receptor antagonist modulating dopaminergic processing, presumably by blocking presynaptic auto‐receptors at lower doses and postsynaptic receptors at higher doses. The current dose of 400 mg is considered an intermediate dose that does not lead to significant sedation or change in subjective alertness, anxiety, restlessness, tiredness, dysphoria nor autonomic disturbances, such as tremor, rigidity, orthostatic hypotension, nausea, or dizziness in healthy volunteers (Mehta et al. 1999; Wiesel et al. 1982). Thus, a single 400 mg dose of sulpiride in healthy volunteers is generally well tolerated, with minimal subjective and autonomic side effects and only occasional mild cognitive slowing. The administration was triple blinded, meaning that neither the participant, the medical personnel, nor the experimenter knew whether placebo or sulpiride pills were administered.

#### 2.4.2 Expectation Manipulation

The same medical doctor or nurse administering the substance also manipulated participants' substance expectation, following a standardized verbal instruction alongside the pill administration. In the high‐expectation condition, participants were told that they had received 400 mg of sulpiride, which would cause short‐term mood enhancements even in non‐depressed individuals. Critically, they were also informed that, although sulpiride is generally safe, it can—like any medication—rarely produce side effects. In the low‐expectation condition, participants were told they had received a placebo pill, with no effects to be expected (see Supporting Information for the verbatim instruction given to the participants). Independent of allocated group, participants were periodically asked whether they experienced any side effects throughout the experiment.

#### 2.4.3 Big Five Aspect Scales—German (BFAS‐G)

Neuroticism was assessed using the German version of the Big Five Aspect Scales (BFAS‐G). The questionnaire consists of 100 items on 7‐point Likert Scales, designed to measure the Big Five personality traits with their respective aspects. The BFAS‐G is a well validated measure, showing convergent validity to the respective scales of other common Big Five measures, like the NEO‐FFI (Borkenau and Ostendorf 2008; Mussel and Paelecke 2018), and exhibiting acceptable to excellent internal consistency for its five main dimensions, coefficient alpha (*α* = 0.71–0.91; Mussel and Paelecke 2018). For this publication only the 20 items measuring Neuroticism were utilized, with 10 items for the volatility aspect and 10 items for the withdrawal aspect.

#### 2.4.4 Generic Rating Scale for Previous Treatment Experiences, Treatment Expectations, and Treatment Effects (G‐EEE)

Initial side‐effect expectations were assessed using the Generic rating scale for previous Treatment Experiences, Treatment Expectations, and Treatment Effects (G‐EEE; Rief et al. 2021). The G‐EEE consists of 10 items that separately assess negative, positive, and side effect expectations, previous experiences, and current effects concerning a specific treatment on 10‐point Likert scales. The G‐EEE is an efficient and valid measure of treatment expectations, as shown by the convergent validity of its respective scales with the longer and well‐established Treatment Expectation Questionnaire (TEX‐Q; Shedden‐Mora et al. 2023). For this publication, only the item assessing side effect expectation was used (“How many complaints/side effects do you expect from the treatment”). Participants had to rate their response on the 10‐point Likert scale from 0 “no complaints” to 10 “strongest complaints imaginable” before the experimental manipulation (see Section 2.3 for details).

#### 2.4.5 General Assessment of Side Effects (GASE)

Side effects were assessed before and after the intervention procedure using the General Assessment of Side Effects questionnaire in the patient version (GASE; Rief et al. 2011, see also Section 2.2). The GASE is a widely used and validated “general screener […] supposed to be used for multiple assessments, to compare side effect profiles at different time points with baseline scores” (Rief et al. 2011, 412). The questionnaire consists of 36 items assessing health complaints across all body regions, including different pain symptoms, nausea, fatigue, and itching. (for a full item‐list, see Rief et al. 2011, 409–410). Items are intended to represent frequently reported side effects observed in clinical trials. Participants had to rate the symptom intensity for each item on a 3‐point Likert scale from 0 “not present” to 3 “severe.”

In the current study, the GASE‐Baseline measure was used to assess general somatic symptoms before the intervention. The GASE‐Post measure was administered after the full procedure and assessed side effects experienced during the period since the intake of the study medication. The interval between the GASE‐Baseline and GASE‐Post measurements was approximately 5.5 to 6 h (see Section 2.2). Before data analysis, we excluded one item measuring “painful or irregular menstruation” as this is a gender specific item. We then calculated a sum score of the remaining 35 items at baseline and postintervention (Rief et al. 2011). GASE‐Baseline was used as a measure of general symptoms. To assess somatic symptoms specific to the intervention procedure, GASE‐Post, with GASE‐Baseline partialled out, was used. As the GASE items assess heterogeneous somatic symptoms across different bodily systems and do not reflect a single latent construct, the total score was treated as a formative symptom index. Therefore, internal consistency estimates (e.g., Cronbach's alpha) were not calculated, consistent with the original validation study (Rief et al. 2011).

### 2.5 Statistical Analysis

Hypotheses were tested in a stepwise fashion. First, the hypothesized bivariate relationships among the investigated variables were tested using bivariate and partial product–moment correlations. In a second step, we used regression models to examine the independent effects of the assumed predictors (neuroticism, side‐effect expectations, and the experimental manipulations) on postintervention symptoms, while controlling for baseline somatic symptoms by including them as a predictor in all regression models. By doing so, we assessed how effect sizes changed when predictors were added sequentially. Finally, the hypothesized mediation was tested using two path models.

The first regression model included neuroticism, baseline somatic symptoms and the experimental manipulation as predictors of postintervention symptoms. In the second model, we added side‐effect expectations as a predictor, investigating how the effect of neuroticism changes by adding pretreatment expectations and whether side‐effect expectations and neuroticism had independent effects on nocebo responses. In a third regression model (see Table S4), we additionally examined possible interaction effects between the interindividual difference variables (neuroticism and side‐effect expectations) and the experimental manipulation.

To test the mediation hypothesis, we used two path models, a partial and a full mediation model. While both models included the same indirect paths from neuroticism via side‐effect expectations and via baseline symptoms to postintervention symptoms, the partial mediation model included an additional direct effect from neuroticism to postintervention symptoms (see Figures 1 and 2). Accordingly, the full mediation model is nested within the partial mediation model.

**FIGURE 1:**
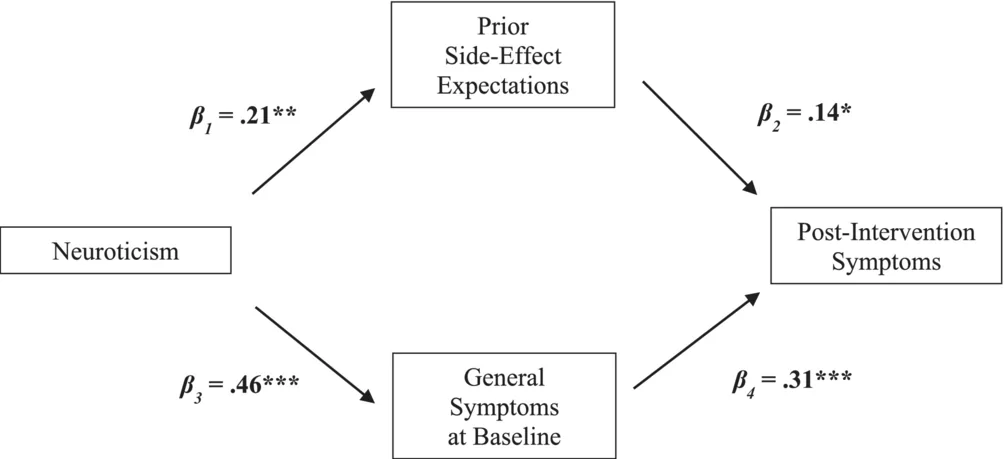
Full mediation model showing standardized paths among neuroticism, initial side‐effect expectations, and postintervention somatic symptoms. Additionally, the model adjusts for baseline general somatic symptoms (with neuroticism predicting these baseline symptoms). *α* = 0.05 (*n* = 275). *p < 0.05; **p < 0.01; ***p < 0.001.

**FIGURE 2:**
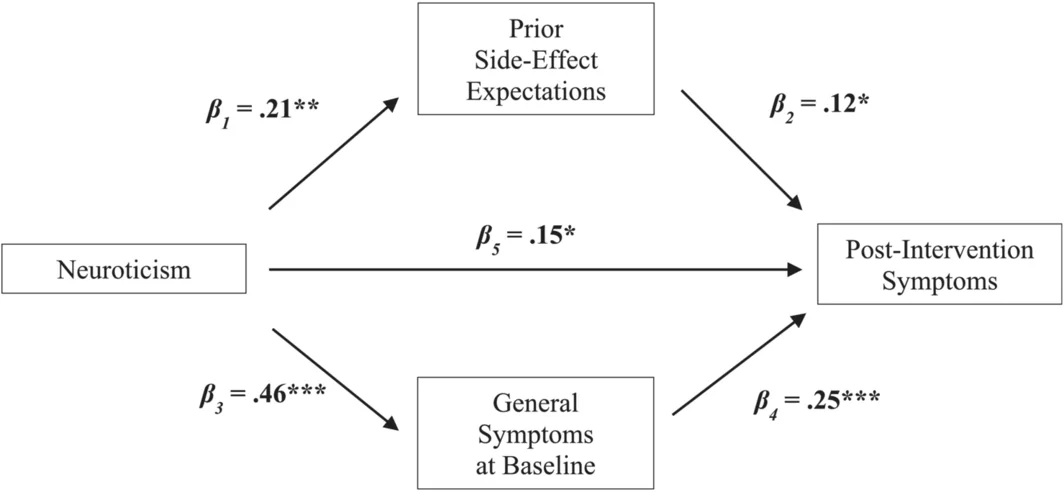
Partial mediation model showing standardized paths among trait neuroticism, initial side‐effect expectations, and postintervention somatic symptoms. The model controls for baseline general somatic symptoms (with neuroticism predicting these symptoms) and, unlike the fully mediated model in Figure 1, includes a direct path from neuroticism to postintervention somatic symptoms. *α* = 0.05 (*n* = 275). *p < 0.05; **p < 0.01; ***p < 0.001.

Both models were evaluated and compared using common fit indices (*χ* ^2^, CFI, RMSEA, SRMR). Potential overfitting of both models was evaluated using 5‐fold cross‐validation and models were directly compared using a *χ* ^2^ difference test, as models are nested. All analyses were conducted in RStudio (R Core Team 2023).

## 3 Results

### 3.1 Bivariate and Partial Correlations

As seen in Table 1, and in line with our first hypothesis, trait neuroticism showed a significant positive correlation with general somatic symptoms at the preintervention baseline (*r* (275) = 0.46, *p* < 0.001, 95% CI = [0.36, 0.55]), with no significant differences between female (*r* (133) = 0.41, *p* < 0.001) and male (*r* (139) = 0.50, *p* < 0.001) participants (*z* = −0.99, *p* = 0.323). Neuroticism was also correlated with postintervention symptoms (*r* = 0.28, *p* < 0.001, 95% CI = [0.17, 0.39]).

**TABLE 1:** Bivariate and partial Pearson correlations among all relevant variables.

	Neuroticism	Symptoms pretreatment	Symptoms posttreatment	Symptoms posttreatment (controlled for pretreatment)
Neuroticism		0.46***	0.28***	0.16**
Side‐Effect Expectations	0.20**	0.12	0.18**	0.15*

*Note:* Correlations are two‐tailed *α* = 0.05 (*n* = 275).

* *p* < 0.05.

** *p* < 0.01.

*** *p* < 0.001.

In accordance with our second hypothesis, the correlation between neuroticism and post‐intervention symptoms remained significant when controlling for baseline symptoms (*r* = 0.16, *p* = 0.009; 95% CI = [0.04, 0.27] see Table 1), showing that individuals with higher neuroticism levels reported more somatic symptoms in general and also reported heightened somatic symptoms following the intervention procedure, independent of their baseline somatic symptom levels. Descriptive statistics for Neuroticism and somatic symptom change scores are provided in Table S3.

A significant association between neuroticism levels and more expected side effects before the intervention (*r* = 0.20, *p* < 0.001; 95% CI = [0.08, 0.31]) confirmed hypothesis 3. Side‐effect expectations were, in turn, significantly correlated with somatic symptoms post intervention (*r* = 0.18, *p* = 0.003, 95% CI = [0.06, 0.29]). Supporting the fourth hypothesis, the correlation between side‐effect expectations and postintervention somatic symptoms remained significant after controlling for baseline symptoms (*r* = 0.15, *p* = 0.012, 95% CI = [0.03, 0.26]).

### 3.2 Regression Analyses

A first regression model (see Table 2) confirmed that postintervention somatic symptoms were independently predicted by trait neuroticism (*β* = 0.17, *p* = 0.008) and general somatic symptoms at baseline (*β* = 0.24, *p* < 0.001), whereas neither the expectation manipulation (*β* = −0.04, *p* = 0.67) nor the substance manipulation (*β* = −0.09, *p* = 0.22) or their interaction (*β* = 0.06, *p* = 0.58) were significant predictors. An additional regression model (see Table S4) found no significant neuroticism × expectation (N × E) or neuroticism × substance (N × S) interactions, indicating that the effect of neuroticism was consistent across experimental conditions.

**TABLE 2:** Independent effects of general somatic symptoms at baseline, trait neuroticism, and experimental manipulations on postintervention symptoms.

Variables	Unstandardized coefficients		Standardized coefficients	*t*	*p*
	*B*	SE	*β*		
Symptoms at baseline	0.15	0.04	**0.24**	3.798***	< 0.001
Trait neuroticism (N)	0.55	0.21	**0.17**	2.680**	0.008
Expectation condition (E)	−0.19	0.46	−0.04	−0.432	0.67
Substance condition (S)	−0.56	0.46	−0.09	−1.221	0.22
E × S	0.36	0.64	0.06	0.561	0.58

*Note:* All regression coefficients were tested for statistical significance using two‐sided tests *α* = 0.05 (*n* = 275).

* *p* < 0.05.

** *p* < 0.01.

*** *p* < 0.001.

When preintervention side‐effect expectations were added as a predictor of post‐intervention symptoms. Side‐effect expectations showed a significant unique effect (*β* = 0.12, *p* = 0.04; see Table 3), while the independent effects of neuroticism (*β* = 0.15, *p* = 0.02) and baseline symptoms (*β* = 0.24, *p* < 0.001) remained significant (see Table 3). Again, no significant interaction effects of side‐effect expectations × substance (S) or side‐effect expectations × expectation (E) were found (see Table S4). Together, this shows that interindividual differences in preintervention side‐effect expectations and neuroticism independently predict reported side effects after the intervention, regardless of the pharmacological treatment, expectation manipulation or baseline symptom levels.

**TABLE 3:** Independent effects of general somatic symptoms at baseline, trait neuroticism, side‐effect expectations and experimental manipulation on postintervention symptoms.

Variables	Unstandardized coefficients		Standardized coefficients	*t*	*p*
	*B*	SE	*β*		
Symptoms at baseline	0.15	0.04	**0.24**	3.731***	< 0.001
Trait neuroticism	0.49	0.21	**0.15**	2.342*	0.02
Prior side‐effect expectations	0.23	0.11	**0.12**	2.091*	0.04
Expectation condition (E)	−0.28	0.45	−0.05	−0.623	0.53
Substance condition (S)	−0.54	0.45	−0.10	−1.182	0.24
E × S	0.35	0.63	0.05	0.548	0.58

*Note:* All regression coefficients were tested for statistical significance using two‐sided tests *α* = 0.05 (*n* = 275).

* *p* < 0.05.

** *p* < 0.01.

*** *p* < 0.001.

### 3.3 Path Analysis

Based on Hypothesis 5, two path models were specified, a full (Figure 1) and a partial (Figure 2) mediation model. Both models postulate that trait neuroticism effects postintervention somatic symptoms via prior side‐effect expectations, include general somatic symptoms at baseline as an independent predictor of post‐intervention symptoms, and allow that trait neuroticism also predicts those baseline somatic symptoms. The partial mediation model includes an additional direct effect of neuroticism on post‐intervention somatic symptoms.

Standardized path coefficients for the full‐mediation model indicated that neuroticism significantly predicted side‐effect expectations regarding the intervention (*β*₁ = 0.21, *p* = 0.001), which in turn significantly predicted postintervention somatic symptoms (*β*₂ = 0.14, *p* = 0.012). Baseline somatic symptoms also independently predicted postintervention symptoms (*β*₄ = 0.31, *p* < 0.001) and were themselves significantly predicted by neuroticism (*β*₃ = 0.46, *p* < 0.001). Finally, the indirect effect of neuroticism on postintervention somatic symptoms via side‐effect expectations, controlling for baseline symptoms, was significant (*β*₁ × *β*₂ = 0.03, *p* = 0.041).

The full mediation model (df = 3) exhibited overall acceptable fit to the data (*χ* ^2^ = 5.392, *p* = 0.067; CFI = 0.970, RMSEA = 0.079, SRMR = 0.036). An additional *k* = 5 cross‐validation showed robust fit indices (CFI = 0.973, RMSEA = 0.054, SRMR = 0.045), suggesting that the model is unlikely to be overfitted and probably generalizes beyond the dataset, even with the relatively low degrees of freedom of the model (see Table 4).

**TABLE 4:** Fit indices and 5‐fold cross‐validation results for the full and partial mediation model.

Fit Measure	Full Mediation Model		Partial Mediation Model	
	Full Sample Fit	5‐Fold Cross Validation	Full Sample Fit	5‐Fold Cross Validation
*χ* ^2^	5.392 (*p* = 0.067)	—	0.270 (*p* = 0.603)	—
CFI	0.970	0.973	1.00	1.00
RMSEA	0.079	0.054	0.000	0.000
SRMR	0.036	0.045	0.009	0.024
Δ *χ* ^2^	—	—	5.12 (Δdf = 1, *p* = 0.024)	—

Abbreviations: CFI, Comparative Fit Index; RMSEA, root mean square error of approximation; SRMR, standardized root mean square residual; Δ*χ* ^2^, difference in *χ* ^2^ between the full and partial mediation models; *χ* ^2^, chi‐squared statistic.

For the partial‐mediation model a direct path from trait neuroticism to postintervention somatic symptoms was added, nesting it within the full‐mediation model. Standardized path coefficients closely mirrored those of the full‐mediation model: neuroticism predicted side‐effect expectations (*β*₁ = 0.21, *p* = 0.001), which in turn predicted postintervention somatic symptoms (*β*₂ = 0.12, *p* = 0.034). Baseline somatic symptoms again independently predicted postintervention symptoms (*β*₄ = 0.25, *p* < 0.001), and neuroticism predicted baseline symptoms (*β*₃ = 0.46, *p* < 0.001).

Notably, the direct standardized path from neuroticism to postintervention somatic symptoms was significant in this partial mediation model (*β*₅ = 0.15, *p* = 0.023). This is in line with the partial correlation (*r* = 0.16, *p* = 0.009) and regression coefficient (*β* = 0.15, *p* = 0.02) reported in Tables 1 and 3. Although the indirect effect via side‐effect expectations (*β*₁ × *β*₂ = 0.03) had almost the same magnitude as in the full mediation model, it did not reach statistical significance in the partial model (*p* = 0.071).

The partial mediation model demonstrated an overall excellent fit (*χ* ^2^ = 0.270, *p* = 0.603; CFI = 1.00; RMSEA = 0.000; SRMR = 0.009). Furthermore, the 5‐fold cross‐validation supported the model's robustness against overfitting (CFI = 1.00; RMSEA = 0.000; SRMR = 0.024; see Table 4).

### 3.4 Direct Model Comparison

*A χ* ^2^ difference test for nested path models directly showed that the partial mediation model fits the data significantly better than the full mediation model (Δ*χ* ^2^ = 5.12, Δdf = 1, *p* = 0.024). Taken together, the fit indices and 5‐fold cross validation and the direct model comparison indicate that the partial mediation model captured the empirical relationships among trait neuroticism, preintervention side‐effect expectations, baseline and postintervention symptoms more neatly than the full mediation model.

## 4 Discussion

The present study investigated whether individual differences in neuroticism and pretreatment side‐effect expectations influence nocebo responses in a pharmacological study with healthy individuals. Specifically, we tested whether such adverse expectations mediate the relationship between neuroticism and nocebo responses.

In doing so, we replicated (1) previous findings demonstrating that neuroticism is robustly associated with generally elevated somatic complaints. We further showed that neuroticism is associated with (2) increased side‐effect expectations prior to the intervention and (3) a heightened nocebo response following the intervention. Moreover, we showed that (4) side‐effect expectations predict nocebo responses and, crucially, that (5) the relationship between neuroticism and heightened nocebo responses is partially mediated by these preintervention expectations.

### 4.1 Neuroticism Relates to Heightened Somatic Symptom Complaints

Regarding general somatic symptoms, we showed that neuroticism strongly predicts somatic symptom reporting prior to the intervention, indicating that individuals with higher neuroticism scores exhibit elevated somatic complaints independent of medical treatments. Although this association has been reported previously (Denovan et al. 2019; Rosmalen et al. 2007), those studies observed smaller effect sizes (*r* = 0.30 and *β* = 0.32, respectively) than ours (*r* = 0.46 with a 95% confidence interval from 0.36 to 0.55). One possible explanation is that earlier work employed narrower symptom measures (the 8‐item Somatic Symptom Scale; Denovan et al. 2019, and the 22‐item symptom checklist; Rosmalen et al. 2007). By contrast, we used the 36‐item Generic Assessment of Side Effects (GASE), a comprehensive multisystem screening tool spanning the medically unexplained–explained continuum (Rief et al. 2011). The use of this broader instrument may thus have led to the inclusion of medically unexplained symptoms with additional variance that could be accounted for by neuroticism.

### 4.2 Neuroticism Relates to Increased Preintervention Side‐Effect Expectations

Neuroticism was also significantly associated with preintervention side‐effect expectations, suggesting that individuals high in neuroticism anticipate more adverse effects even before treatment begins. This aligns with high‐neuroticism individuals exhibiting heightened negative expectations in general (Fischer and Mueller 2025). Notably, the correlation between neuroticism and specific side‐effect expectations was weaker than that for broad life‐outcome expectations, consistent with the bandwidth–fidelity dilemma (Cronbach and Gleser 1957). This is expected because situational factors play a pivotal role in determining expectations in individual medical interventions (Rief and Petrie 2016), thereby limiting the variance explained by broad traits, whereas situational influences average out for broad life‐outcome expectations. The observed link between neuroticism and intervention‐specific side‐effect expectations is also consistent with the prior report on a partially overlapping sample of *n* = 748 participants (Basedow et al. 2023), although the correlation was smaller in that prior multi‐site study, presumably due to a different statistical approach involving several intercorrelated predictors in a single regression model (Basedow et al. 2023). Together, these findings suggest that neuroticism is not only characterized by generalized negative expectations but also by very specific side‐effect expectations prior to a single intervention.

### 4.3 Neuroticism and Side‐Effect Expectations Predict Nocebo Responses Independently

Of particular relevance, we demonstrated that both neuroticism and preintervention side‐effect expectations independently and significantly predict a heightened nocebo response after a single intervention.

In terms of neuroticism, our large sample (*n* = 275) afforded sufficient statistical power to roughly estimate the magnitude of the small association between neuroticism and a heightened nocebo response (*r* = 0.16, 95% CI [0.04, 0.27]). Note that even for a correlation of *r* = 0.27, which is the upper bound of our estimated confidence interval, a sample size of *n* = 83 would be required to achieve a power of 0.8. For a true correlation of *r* = 0.16, a sample size of *n* = 240 would be needed. Clearly, the majority of studies on individual differences in nocebo responses would be underpowered to detect associations of this size (for review see Kern et al. 2020).

The correlation between side‐effect expectations and a heightened nocebo response was also significant. In light of the central role of treatment expectations in nocebo phenomena (Rief and Petrie 2016), the magnitude of this correlation (*r* = 0.15) may appear comparably low. It should be noted, however, that side‐effect expectations had been assessed with a single item measure in this study, thereby reducing the reliability and consequently, the upper bound of any to‐be‐expected predictor–criterion correlations. Moreover, it should be noted that neither the experimental expectation manipulation nor the actual pharmacological substance had an impact on reported side effects, suggesting that the overall level (and consequently, interindividual variability) of side effects was rather low, thereby further working against high correlations between side‐effect expectations and actual side‐effect experiences.

Further note that neither the substance nor the expectation manipulation influenced the effects of neuroticism or side‐effect expectations on nocebo responses. This absence is reasonable given the lack of substantial side effects following a single 400 mg dose in healthy volunteers (Mehta et al. 1999, 2008; Wiesel et al. 1982) and the fact that the expectation manipulation was not primarily intended to induce side effects. Participants were simply informed of their alleged allocation (sulpiride vs. placebo; see Section 4.6) and were periodically asked whether they experienced any side effects throughout the experiment, regardless of group allocation.

Taken together, these findings show that trait neuroticism and pretreatment side‐effect expectations uniquely predict a heightened nocebo response, independent of experimental manipulations, likely by biasing individuals to interpret any somatic experience during the intervention as more adverse. This adverse interpretive bias aligns with the well‐documented negative cognitive‐emotional biases associated with neuroticism (Suls and Martin 2005) and underscores the importance of interindividual trait and expectation differences as predictors of nocebo responses, even after single interventions.

### 4.4 The Effects of Neuroticism on Nocebo Responses Is Partially Mediated by Side‐Effect Expectations

The key integrative finding of the current study is that preintervention side‐effect expectations partially mediate the link between neuroticism and a heightened nocebo response. This finding, derived from comparing a full and a partial mediation model, integrates the above‐reported effects into a single framework, providing a mechanistic understanding of the neuroticism–nocebo response link.

With regard to the *indirect* effect of neuroticism on nocebo responses, mediated through side‐effect expectations, it can be assumed that (A) higher neuroticism leads to increased side‐effect expectations, as a situation‐specific manifestation (Panitz et al. 2021) of broad negative expectations in neuroticism more generally (Fischer and Mueller 2025) and that (B) increased side‐effect expectations are then the primary driver of nocebo responses (Rief and Petrie 2016). This indirect path thus illustrates how a neuroticism‐associated negative outlook translates into nocebo responses via explicit side‐effect expectations.

With regard to the *direct* effect of neuroticism on heightened nocebo responses, it should be noted that there are information processing biases in high neuroticism that may affect nocebo responses above and beyond the explicit side‐effect expectations that a participant can report in a single item. These may include negative attributional and interpretational biases or somatosensory amplification (Suls and Martin 2005; Wise and Mann 1994), which are themselves increasingly seen as less consciously accessible negative expectancies that skew the interpretation of incoming interoceptive and exteroceptive signals (Seth and Friston 2016; Van Den Bergh et al. 2017).

In sum, the evidence for a partial mediation model indicates that neuroticism drives nocebo responses both indirectly, by heightened, consciously held side‐effect expectations, and directly, presumably by negatively biasing information processing during the intervention. To our knowledge, the current study is the first experimental study to integrate these effects of neuroticism and side‐effect expectations as predictors of nocebo responses, highlighting the importance of individual differences for nocebo responses, even after single interventions.

### 4.5 Implications

The current findings on the neuroticism–nocebo link, investigating side‐effect expectations as an intermediary, carry both theoretical and practical implications. From a theoretical standpoint, the results fit with emerging predictive‐processing accounts of interoception (Seth and Friston 2016; Van Den Bergh et al. 2017) and personality (Safron and DeYoung 2021). From this lens, high trait neuroticism can be viewed as a variation of a generative model dominated by negative expectations, which are reinforced by maladaptive negative cognitive biases (Fischer and Mueller 2025; Suls and Martin 2005). Interoceptive signals are likewise distorted by negative interoceptive interpretations, such as heightened engagement with adverse bodily sensations (Denovan et al. 2019). This may be indicative of a self‐reinforcing feedback loop characteristic of neuroticism: negative expectations amplify both general somatic complaints and those that arise after medical interventions, which in turn reinforce side‐effect expectations about future treatments, further intensifying nocebo responses in subsequent medical encounters. Of relevance, the combination of similar feedback loops outside the realm of medical treatments and health (e.g., in social cognition, self‐perception, etc.) may contribute to the stability of generalized negative expectations and of neuroticism more generally.

In terms of practical relevance, this paper represents an initial step toward individualizing medical treatments by accounting for interindividual differences in nocebo mechanisms. Even though the association between neuroticism and heightened nocebo responses in this intervention may appear modest, its real‐world impact is likely amplified, as individuals high in neuroticism experience more somatic complaints in general (as shown in this study) and visit medical practitioners more often (Willroth et al. 2023). Consequently, individuals high in neuroticism are more likely to experience cumulative nocebo responses over the course of multiple interventions. This cumulative impact across a series of treatments is likely substantially greater than that observed our single intervention, consistent with the aforementioned bandwidth‐fidelity dilemma (Cronbach and Gleser 1957). In the long run, identifying broad dispositional traits and treatment expectations as predictors of amplified nocebo responses may therefore enable us to mitigate adverse effects for at risk individuals following medical interventions (Nestoriuc et al. 2016). In day‐to‐day clinical practice for example, healthcare providers could identify at‐risk individuals by briefly screening for the mediator identified in this study: specific pretreatment side‐effect expectations (e.g., “Are you currently worried about potential side effects?”). Furthermore, since comprehensive personality testing is impractical, clinicians should remain attentive to behavioral indicators of high neuroticism, such as generalized health anxiety or a history of nonspecific somatic complaints, which we found to be strongly correlated with neuroticism (see also Colloca and Barsky 2020).

For these at‐risk patients, a tailored communication style may most likely be beneficial. This could include positive but accurate framing of healthcare information, where the safety rather than the risk of the drug is emphasized (e.g., ‘90% of patients tolerate this well’). Research indicates that such positive framing can significantly reduce short‐term nocebo effects without providing inaccurate information (Faasse et al. 2019). Additionally, clinicians may employ psychoeducation or “contextualized informed consent” to prevent patients from misinterpreting normal somatic fluctuations as drug‐induced adverse events. By targeting expectations through these strategies, clinicians may interrupt a reinforcing feedback loop between neuroticism and heightened nocebo responses (Colloca and Barsky 2020; Faasse et al. 2019).

### 4.6 Limitations

Despite these theoretical and practical implications, several limitations warrant consideration. First, although we detected a measurable impact of side‐effect expectations on a heightened nocebo response, our brief expectations measure likely reduced reliability and thus may have attenuated the observed effect sizes. We thus cannot rule out that a more comprehensive measure of side effect expectations might have led to a higher correlation between side‐effect expectations, neuroticism, and side effect experience and thereby would have provided support for a full mediation model.

Second, the study was not specifically designed to probe nocebo phenomena: As stated above, the expectation manipulation merely informed participants of their alleged allocation (high‐ vs. low‐expectation) without explicitly inducing side‐effect expectations, and a single 400 mg sulpiride dose in healthy volunteers typically elicits no subjective side effects beyond placebo (Mehta et al. 1999, 2008). This approach may have led to smaller nocebo responses than a manipulation in which potential side effects were deliberately induced (i.e., active placebo) or made more salient. On the other hand, this approach may better represent current practice where medical staff spends little time on explaining potential side effects. Moreover, the fact that neuroticism and side‐effect expectations predicted nocebo responses independent of experimental manipulations underscores the importance of individual differences variables for nocebo responses.

Third, our design employed a single, standardized intervention within a controlled biopsychological paradigm, which likely constrained effect sizes relative to those observed in naturalistic physician–patient encounters. Future research in real‐world clinical settings, using longitudinal tracking of multiple visits, would not only enhance ecological validity but also clarify the cumulative impact of neuroticism on an overall nocebo effect.

## 5 Conclusion

In sum, the present study demonstrates that neuroticism is associated with heightened somatic complaints in general and a heightened nocebo response after a psycho‐pharmacological intervention in particular. This link between neuroticism and nocebo responses is partially mediated by increased explicit preintervention side‐effect expectations.

Overall, the present findings represent an initial step toward a more integrative understanding of how stable interindividual differences in negative affect and associated expectations contribute to nocebo responses, paving the way for medical treatments that are personalized according to psychological characteristics. At a broader level, these findings suggest that neuroticism fosters negative expectations, which amplify aversive experiences and, in turn, reinforce both those expectations and negative affect, creating self‐reinforcing feedback loops in individuals high in neuroticism.

## Author Contributions

**Anton Fischer:** writing – original draft, formal analysis, visualization, conceptualization. **Li‐Ching Chuang:** investigation, conceptualization, writing – review and editing. **Nick Augustat:** investigation, conceptualization. **Pia von Blanckenburg:** writing – review and editing. **Erik M. Mueller:** conceptualization, methodology, supervision, writing – review and editing.

## Funding

This work was supported by Deutsche Forschungsgemeinschaft, 290878970‐RTG 2271, 422744262‐TRR289.

## Conflicts of Interest

The authors declare no conflicts of interest.

## Supporting information


**Data S1:** jopy70056‐sup‐0001‐DataS1.docx.

## Data Availability

Data, codebook, and R‐code can be accessed here: https://osf.io/npa8c/overview?view_only=4f37edae9ad44c179ebb0025ed866a67
