# Supplementary material for: Trait Neuroticism and the Nocebo Effect: The Mediating Role of Side‐Effect Expectations
Source: J Pers. 2026 Feb 14;94(6):1006–16. doi: 10.1111/jopy.70056 (PMC13768720; doi:10.1111/jopy.70056)
Supplement: Supplementary file 1 [file JOPY-94-1006-s001.docx]

**Supplementary Material and Methods**

**Detailed Criteria for Participant Inclusion and Exclusion**

In addition to the summary provided in the main text, we present all participant eligibility criteria and statistical exclusion criteria in two separate tables S1 and S2 to enhance transparency regarding participants characteristics and statistical decision making.

**Table S1:** *Participant Eligibility Criteria*

| **Criterion** | **Inclusion** | **Exclusion** |
| --- | --- | --- |
| Demographic | Age 18–65 years; Right‑handed; Informed consent | Pregnancy (positive rapid test) |
| Medical history | — | Endocrinopathies; epilepsy; hypertension; coronary heart disease; peptic ulcer disease or gastrointestinal bleeding; past liver or kidney disease |
| Medication use | — | Any prescription drugs within the last 3 months |
| Anthropometric | — | BMI < 19 or > 30 kg/m² |
| Psychiatric status | — | Suspected DSM‑IV Axis I disorders (Mini‑DIPS screening for affective, somatoform, psychotic, anxiety, eating, adjustment, and substance‑related disorders) |
| Lifestyle factors | — | Regular cigarette smoking; illicit substance use; alcohol abuse; high caffeine consumption (> 8 cups/day); proficiency in Japanese (per learning‑task requirements) |

**Table S2:** Post‑Screening Statistical Exclusions

| **Exclusion Type** | **Criterion** |
| --- | --- |
| Missing data | Any missing values in assessed variables |
| Outliers | Observations > 3 × IQR in any variable |

*Note.* IQR = interquartile range

**Descriptive Statistics for Neuroticism and Somatic Symptom Changes**

**Table S3:** *Descriptive Statistics for Neuroticism and Somatic Symptom Change Scores*

| **Variable** | **Mean (*M)*** | **Standard Deviation (*SD)*** |
| --- | --- | --- |
| Neuroticism (BFAS-N) | 3.24 | 0.88 |
| GASE Change Score (Post – Baseline) | -1.44 | 4.53 |

The negative mean change score in the GASE indicates a general decrease in reported symptoms. This is likely due to the different reference frames: Baseline measurements captured general somatic complaints, while the post-measurement focused specifically on new symptoms occurring during the intervention period.

**Verbatim Instructions for the Expectation Manipulation Provided to Participants**

***Sulpiride Expectation Manipulation***

“Dear Ms./Mr. [Name of participant],
I’m pleased to meet you and would first like to thank you for taking part in our study. My name is [Name of administering staff] and I am responsible for the medical supervision of this study. First, I can tell you that you have been assigned to the medication group rather than the placebo group by random draw, and will therefore shortly receive a very effective drug that should have a positive effect on your mood during the study. The medication you will receive is sulpiride in a dose of 400 mg. Sulpiride is an approved medication in Germany and belongs to the class of antidepressants. We use it, for example, in people suffering from depressive disorders. Even a single tablet produces a noticeable improvement in mood—in other words, you feel much more cheerful and experience greater joy. What’s special is that the mood-lifting effect also develops in people who are not suffering from depression. Sulpiride is a very safe and well-tolerated drug. As with any other preparation, sulpiride can, in rare cases, cause side effects. So if you experience anything unpleasant, just let us know. It takes about three hours for sulpiride to reach its full effect in the brain. You should then notice that your mood is significantly improved for the duration of the experiment. If you have any questions, please feel free to ask them at any time.”

***Placebo Expectation Manipulation***

“Dear Ms./Mr. [Name of participant],
I’m pleased to meet you and would first like to thank you for taking part in our study. My name is [Name of administering staff] and I am responsible for the medical supervision of this study. First, I can tell you that you have been assigned to the placebo group rather than the medication group by random draw. This means that you will shortly receive an inert capsule as a sham medication. However, this capsule contains no active ingredient and therefore has no effect on your body while you participate in the study. If you have any questions, please feel free to ask them at any time.”

**Interaction Effects of Interindividual Difference Variables and Experimental Manipulations on Post-Intervention Symptoms**

In this additional analysis (Table S3), we examined whether the effects of the examined interindividual difference variables (trait neuroticism (N) and side-effect expectations) were moderated by the experimental manipulations (Expectation (E) and Substance (S)). No significant interaction effects were observed, suggesting that the influence of neuroticism and side-effect expectations on post-intervention symptoms were independent of the experimental manipulations. This finding was to be expected, as the manipulations were not primarily intended to target nocebo responses (see main text for further discussion).

| Variables | Unstandardized  Coefficients | | Standardized Coefficients |  |  |
| --- | --- | --- | --- | --- | --- |
|  | *B* | *SE* | *β* | *t* | *p* |
| Expectation condition (E) | .62 | 1.22 | **.11** | .51 | .61 |
| Substance condition (S) | .68 | 1.22 | **.12** | .56 | .58 |
| Baseline Symptoms | .15 | .04 | **.24** | 3.80*** | < .001 |
| Prior Side-Effect Expectations | .31 | .21 | **.16** | 1.46 | .14 |
| Trait Neuroticism | .73 | .36 | **.23** | 2.05* | .04 |
| Prior Side-Effect Expectation x E | .10 | .23 | **.05** | .428 | .67 |
| Prior Side-Effect Expectations x S | -.29 | .22 | **-.12** | -1.320 | .18 |
| Neuroticism x E | -.25 | .38 | **-.16** | -.677 | .50 |
| Neuroticism x S | -.22 | .37 | **-.14** | -.585 | .56 |

**Table S4:**
Main and Interaction Effects of all Inter-individual Difference Variables and Experimental Manipulations on Post-Intervention Symptoms.

*Note.* All regression coefficients were tested for statistical significance using two-sided tests *α* = .05 (*n* = 275).
* *p* < .05; ** *p* < .01; *** *p* < .001
